# Isolation and characterization of a novel *Staphylococcus* phage SPD: genomic insights and its anti-biofilm efficacy against methicillin-resistant *Staphylococcus aureus*

**DOI:** 10.3389/fcimb.2026.1861467

**Published:** 2026-08-12

**Authors:** Xiaofeng Zheng, Lan Ma, Yongchao Li, Bo Liu, Wei Zhang, Xiaoyue Su, Yingyu Liu

**Affiliations:** 1 College of Veterinary Medicine, Xinjiang Agricultural University, Urumqi, China; 2 Xinjiang Key Laboratory of New Drug Research and Development for Herbivorous AnimaIs (XJ-LNDRDHA), Urumqi, China; 3 Urumqi Field Scientific Observation and Research Station for Animal Diseases, Ministry of Agriculture and Rural Affairs, Urumqi, China; 4 Xinjiang Regional Key Laboratory of Clinical Veterinary Medicine Research (XJRKLCVMR), Urumqi, China; 5 Herbivorous Animal Biomedicine and Diagnostic Technology Engineering Research Center, Urumqi, China; 6 College of Veterinary Medicine, Nanjing Agricultural University, Nanjing, China; 7 The Sanya Institute of Nanjing Agricultural University, Sanya, China; 8 Key Lab of Animal Bacteriology, Ministry of Agriculture, Nanjing, China; 9 Bayinguoleng Vocational and Technical College, Bayinguoleng, Xinjiang, China

**Keywords:** *Andhravirus*, bacteriophage, biofilm, genomics, methicillin-resistant *Staphylococcus aureus* (MRSA)

## Abstract

**Objective:**

To isolate and characterize a novel *Staphylococcus* phage with activity against animal-derived methicillin-resistant *Staphylococcus aureus* (MRSA) infections and biofilms in Kashgar, Xinjiang.

**Methods:**

Phage SPD was isolated through enrichment from farm wastewater, purified using the double-layer plaque assay, and characterized by using transmission electron microscopy (TEM), spot assays, growth kinetics, stability tests, and whole-genome sequencing (Illumina HiSeq). Anti-biofilm activity was evaluated via crystal violet staining.

**Results:**

SPD is a virulent *Podovirus* characterized by an icosahedral head (~50 nm in diameter) and a short tail phage (~60 nm in length). It exhibits a broad host range (lyses 38 out of 50 tested strains, including 7 MRSA), a short latent period (~10 min), a high burst size (4,470 PFU/cell), and stability at temperatures ranging from 4 to 50 °C and pH values between 6 and 9. The 17,066 bp double-stranded DNA genome (35.1% GC content) encodes a lysin and a holin, with no virulence or antibiotic resistance genes detected. SPD effectively inhibits planktonic MRSA growth and reduces biofilm formation (>50% inhibition) as well as pre-formed biofilms (>60% clearance) *in vitro*.

**Conclusion:**

SPD represents a safe, novel *Andhravirus* phage with potent activity against MRSA and biofilms, offering a promising candidate for phage therapy, particularly in the Xinjiang region.

## 1 Introduction

*Staphylococcus aureus* is a major pathogen responsible for both community- and hospital-acquired infections, causing a wide spectrum of diseases, including skin and soft tissue infections, pneumonia, bacteremia and osteomyelitis. This poses a serious challenge to global public health (Duarte et al., 2025; Wang et al., 2026; Zhang et al., 2026). The emergence and widespread dissemination of MRSA are particularly concerning, as its resistance to multiple β-lactam antibiotics severely limits clinical treatment options and leads to significantly increased patient mortality (Liu et al., 2026; Zhang et al., 2026). Moreover, *S*. aureus can form biofilms, which are structures that substantially enhance bacterial resistance to antibiotics and host immune clearance. Biofilms serve as a key factor in chronic, recurrent infections and medical device-associated infections (Garcia-de-Albeniz et al., 2026; Santonocito et al., 2026). Given the inefficacy and high recurrence rates of conventional antibiotic treatments for MRSA biofilm infections, the development of novel and effective anti-infective strategies is urgently needed.

Bacteriophages (phages) are viruses that specifically infect and lyse bacterial cells. First, lytic phages exhibit high host specificity, which allows them to selectively eliminate pathogenic bacteria without disrupting the commensal microbiota—a critical benefit for maintaining gut and skin homeostasis during infection treatment (Pye et al., 2024; Doud et al., 2025). Second, phages can self-amplification at the site of infection, achieving exponential increases in therapeutic dose through *in situ* replication, which is particularly advantageous for penetrating biofilms and reaching deep tissue infections (Wang et al., 2024). Third, phages and their derived enzymes, such as endolysins and depolymerases, have demonstrated efficacy in degrading the biofilm extracellular polymeric matrix, a feature that directly addresses the challenge of chronic and device-associated MRSA infections (Ceballos-Zuniga et al., 2025; Zhu et al., 2026). Despite the promising prospects of phage therapy, its efficacy is highly dependent on the specificity of phage-host strain matching. Therefore, the continuous isolation, identification, and characterization of novel phages with broad lytic activity-particularly those targeting prevalent MRSA strains from specific geographical regions or host sources-are essential for advancing phage therapy toward clinical application.

Xinjiang, a region with a well-developed livestock industry and frequent human-animal contact, provides a unique environment for the transmission and evolution of zoonotic pathogens, including MRSA. Recent surveillance studies have revealed that the predominant MRSA lineages circulating in this region belong to ST9-t899 and ST398-t034, with the latter being frequently associated with pig and pigeon farms (Wang et al., 2015). However, research on phage resources that target prevalent MRSA strains in this region remains limited. In this study, we isolated a novel *Staphylococcus* phage, designated SPD, from the environment of a pigeon farm in Kashgar, Xinjiang. This study aimed to systematically characterize its morphology, biological properties (host range, stability, one-step growth curve), whole-genome features (genomic structure, functional annotation, phylogenetic position), and *in vitro* antibacterial activity against both planktonic MRSA and biofilms. The findings aim to enrich the *Staphylococcus* phage resource library in China and provide important experimental evidence for evaluating SPD as a potential therapeutic agent against MRSA infections, particularly those involving biofilms.

## 2 Materials and methods

### 2.1 Bacterial strains

All *Staphylococcus aureus* strains used in this study were previously isolated, identified, and preserved by the Laboratory of Animal Product Quality and Safety, College of Veterinary Medicine, Xinjiang Agricultural University. Initial identification was performed using standard biochemical tests, including Gram staining, catalase test (3% H_2_O_2_), and tube coagulase test (rabbit plasma). To identify methicillin-resistant S. aureus (MRSA) strains, PCR amplification of the *mecA* gene was performed using the following primers: forward primer F (5’-ACCAGATTACAACTTCACCAGG-3’) and reverse primer R (5’-CCACTTCATATCTTGTAACG-3’), yielding an amplicon of 164 bp. The PCR conditions were as follows: initial denaturation at 94 °C for 5 min, followed by 30 cycles of 94 °C for 30 s, 55 °C for 30 s, and 72 °C for 30 s, with a final extension at 72 °C for 5 min. All primers were synthesized by Shanghai Sangon Biotech Co., Ltd. MRSA strain 380-2, which was isolated from a pigeon farm in Kashgar, Xinjiang, China, served as the host strain for phage isolation. To determine the host range of phage SPD, a total of 50 bacterial isolates were used, comprising 41 *Staphylococcus aureus* strains, 7 MRSA strains, and 2 *Staphylococcus lentus* strains (**Table 1**). All strains were cultured overnight in broth medium supplemented with 7.5% sodium chloride at 37 °C with shaking at 180 rpm.

**Table 1:** Host range of SPD phages.

Strain number	Source	Species	SPA typing	Phage lytic ability of bacteria
SAN6-2	pigeon	*Staphylococcus aureus*	T002	–
238-2	pigeon	*Staphylococcus aureus*	t091	+
CZT-8	pigeon	*Staphylococcus aureus*	t091	+
SA4-1	pigeon	*Staphylococcus aureus*	t1059	+
SAN4-3	pigeon	*Staphylococcus aureus*	t127	+
55-2	pigeon	*Staphylococcus aureus*	t127	+
71-1	pigeon	*Staphylococcus aureus*	t127	+
90-1	pigeon	*Staphylococcus aureus*	t127	+
381-2	pigeon	*Staphylococcus aureus*	t127	+
384-2	pigeon	*Staphylococcus aureus*	t127	+
386-2	pigeon	*Staphylococcus aureus*	t127	+
SAH2-1	pigeon	*Staphylococcus aureus*	t1670	+
103-1	pigeon	*Staphylococcus aureus*	t2484	+
104-1	pigeon	*Staphylococcus aureus*	t2484	+
245-1	pigeon	*Staphylococcus aureus*	t2484	+
SAM1-2	pigeon	*Staphylococcus aureus*	t267	+
101-1	pigeon	*Staphylococcus aureus*	t267	+
130-1	pigeon	*Staphylococcus aureus*	t309	+
131-1	pigeon	*Staphylococcus aureus*	t309	+
SAN5-1	pigeon	*Staphylococcus aureus*	t4792	+
113-1	pigeon	*Staphylococcus aureus*	t7229	+
511-1	pigeon	*Staphylococcus aureus*	t803	–
CZT-8	goose	*Staphylococcus aureus*	t091	+
TTGR-1	goose	*Staphylococcus aureus*	t078	–
D64	cattle	*Staphylococcus aureus*	t267	+
N16	cattle	*Staphylococcus aureus*	t2616	+
D65	cattle	*Staphylococcus aureus*	t1328	+
D82	cattle	*Staphylococcus aureus*	t127	+
N1	cattle	*Staphylococcus aureus*	unknown	+
D68	cattle	*Staphylococcus aureus*	t2592	–
143	donkey	*Staphylococcus aureus*	unknown	+
150	donkey	*Staphylococcus aureus*	t2484	–
D337	sheep	*Staphylococcus aureus*	t1736	+
6-14	sheep	*Staphylococcus aureus*	t127	–
129	sheep	*Staphylococcus aureus*	t078	–
46	pig	*Staphylococcus aureus*	t189	+
6-57	pig	*Staphylococcus aureus*	t437	–
79	pig	*Staphylococcus aureus*	t189	–
A41	camel	*Staphylococcus aureus*	t435	–
A202	chicken	*Staphylococcus aureus*	t127	–
A269	chicken	*Staphylococcus aureus*	t8891	–
378-1	pigeon	MRSA	t034	+
380-2	pigeon	MRSA	t034	+
390-1	pigeon	MRSA	t034	+
393-1	pigeon	MRSA	t034	+
404-1	pigeon	MRSA	t034	+
408-1	pigeon	MRSA	t034	+
415-2	pigeon	MRSA	t034	+
WH61	unknown	*Staphylococcus lentus*		+
WE17	unknown	*Staphylococcus lentus*		+

### 2.2 Spa typing

Spa typing was performed following the method described by Liu et al (Liu et al., 2021). The primers used for amplification of the spa gene polymorphic X region were spa-F (5’-AGACGATCCTTCGGTGAGC-3’) and spa-R (5’-GCTTTTGCAATGTCATTTACTG-3’), producing amplicons of approximately 350–450 bp. PCR products were purified and sequenced by Sanger sequencing at Beijing Tsingke Biotechnology Co., Ltd. (Xi’an Branch). The obtained sequences were submitted to the CGE Server spa typing tool (https://cge.food.dtu.dk/services/spaTyper/) for spa type assignment based on the Ridom nomenclature.

### 2.3 Phage isolation, purification, and propagation

Phage isolation samples were collected from sewage at a pigeon farm in Kashgar, Xinjiang, China. The samples were centrifuged at 5,000 rpm for 10 min, and the supernatant was sterilized by filtration through a 0.22 µm membrane filter. One milliliter of the filtrate was mixed with 1 mL of host bacteria (MRSA 380-2) in the logarithmic growth phase and inoculated into 10 mL of LB broth, followed by incubation at 37 °C with shaking at 180 rpm for 18 h. After centrifugation at 6,000 rpm for 10 min, the supernatant was filtered again through a 0.22 µm membrane. The resulting filtrate was the phage enrichment solution. Phage purification was performed using the double-layer agar method (Ma et al., 2025). Briefly, 100 µL of phage enrichment solution was mixed with 100 µL of logarithmic-phase host bacteria and adsorbed at room temperature for 10 min. The mixture was then combined with 7 mL of semi-solid LB agar (0.5% agar) and overlaid onto solidified LB solid agar (1.5% agar) plate. After overnight incubation at 37 °C, individual plaques with uniform morphology were picked and purified by repeating the above steps 3–5 times. The purified phage was designated SPD and stored at 4 °C in SM buffer (pH 7.5, 6.1 g/L Tris-HCl, 0.1 g/L Gelatin, 2g/L MgSO_4_·7H_2_O, and 5.8 g/L NaCl) for subsequent use.

### 2.4 Transmission electron microscopy of phage

The purified phage SPD was filtered through a 0.22 µm membrane, and the resulting sterile phage lysate was sent to Wuhan Servier Company for analysis. Following negative staining with phosphotungstic acid, the morphological structure of the phage was observed and imaged using transmission electron microscopy (Zheng et al., 2024).

### 2.5 Determination of phage host range

The host range of phage SPD was determined using the spot assay (Zheng et al., 2025). Logarithmic-phase cultures (100 µL) of each test strain were mixed with 7 mL of semi-solid LB agar and overlaid onto LB agar plates to form a bacterial lawn. After the top agar solidified, 7 µL of phage SPD enrichment solution was spotted onto the surface and allowed to absorb at room temperature. The plates were then incubated overnight at 37 °C, and the formation of clear plaques was observed and recorded. Strains exhibiting clear plaques were considered susceptible.

### 2.6 Determination of optimal multiplicity of infection

The phage SPD suspension was serially diluted in SM buffer and mixed with logarithmic-phase host bacteria (MRSA 380-2) at MOI ratios of 10, 1, 0.1, 0.01, 0.001, and 0.0001. A 100 µL aliquot of each mixture was combined with 100 µL of host bacteria, adsorbed at room temperature for 10 min, and then supplemented with 4 mL of LB broth, followed by incubation at 37 °C with shaking at 180 rpm for 4 h. After incubation, samples were immediately taken and phage titers were determined using the double-layer agar method. The MOI yielding the highest phage titer was identified as the optimal MOI.

### 2.7 Thermal and pH stability of phage

To assess thermal stability, 500 µL aliquots of phage SPD suspension were incubated in water baths at 4, 30, 40, 50, 60, and 70 °C. Samples were collected at 20, 40, and 60 min, and phage titers were immediately determined using the double-layer agar method.

To evaluate pH stability, 100 µL of phage SPD suspension was added to 900 µL of LB broth adjusted to different pH values (2, 3, 4, 5, 6, 7, 8, 9, 10, 11, 12, and 13) and incubated statically at 37 °C for 1 h. Phage titers were then determined using the double-layer agar method.

### 2.8 One-step growth curve of phage

Phage SPD and host bacteria were mixed at the optimal MOI and adsorbed at room temperature for 10 min (Zhang et al., 2017). The mixture was then centrifuged at 6,000 rpm for 10 min, and the supernatant was discarded. The pellet was washed twice with phosphate buffered saline (PBS) and resuspended in 4 mL of LB broth, followed by incubation at 37 °C with shaking at 180 rpm. Samples were collected every 10 min over a period of 120 min, and phage titers were determined using the double-layer agar method. The burst size was calculated as the ratio of the phage titer at the end of the burst phase to the initial bacterial concentration, expressed as PFU/cell.

### 2.9 Whole-genome sequencing and bioinformatics analysis of phage

Genomic DNA was extracted from the phage lysate using the Tiangen Viral DNA/RNA Extraction Kit. The DNA sample was submitted to Tianjin Novogene Bioinformatics Technology Co., Ltd. for library construction and paired-end sequencing on the Illumina HiSeq platform. Raw sequencing data were quality-assessed using FastQC (v0.11.9), and low-quality sequences as well as host-derived reads were filtered out to obtain high-quality clean data. Sequence assembly was performed using Unicycler (v0.0). Automated annotation of the assembled genome was conducted using Prokka (v0.0). The annotated GenBank file was uploaded to Proksee (https://proksee.ca/), where its database (GC Content, GC Skew, Phigaro, etc.) was used to manually manage the genetic map and visualize the gene composition diagram. BLASTp comparisons were performed for phage genomic data, and lysin fragments identified through alignment were analyzed using the SMART online tool for domain prediction. Additionally, phage genomic data were uploaded to the Phagescope platform (https://phagescope.deepomics.org/) to predict the phage lifestyle and screen for anti-CRISPR genes and antimicrobial resistance genes. The presence of virulence factors was analyzed using the Virulence Factors Database (VFDB).

The amino acid sequence of the major capsid protein of phage SPD was compared with homologous sequences using BLASTp. A phylogenetic tree was constructed using MEGA 7.0 software based on the neighbor-joining method with the Poisson correction model, and bootstrap values were calculated from 1,000 replications. The phylogenetic tree was visualized using the EvolView online tool (https://evolgenius.info/). Reference phages with the highest whole-genome similarity to SPD were selected for genome collinearity comparison using Easy Fig 2.2.5 software with the tBLASTx alignment mode.

### 2.10 *In vitro* antibacterial assays of phage

#### 2.10.1 Antibacterial activity against planktonic bacteria at different MOIs

The inhibitory effect of phage SPD on the planktonic growth of 12 host strains, including MRSA 380-2, was measured using a microplate reader. Logarithmic-phase host bacteria were mixed with phage SPD suspension at MOIs of 1, 10, and 100, added to 5 mL of LB broth, and incubated at 37 °C with shaking at 180 rpm. Cultures containing only LB broth served as blank controls. The optical density at 600 nm (OD_600_) of the cultures was measured every 20 min for 6 h using a microplate reader. Cultures without phage served as the untreated control group. Each experiment was performed in triplicate.

#### 2.10.2 Inhibition of biofilm formation

The inhibition of biofilm formation by phage was assessed using crystal violet staining as previously described (Mulani et al., 2022). Logarithmic-phase MRSA cultures were diluted to 10^6^ CFU/mL in TSB and 100 µL was added to each well of a 96-well cell culture plate. An equal volume of phage SPD suspension at different MOIs (1, 10, and 100, diluted in SM buffer) was then added. Positive controls (bacterial suspension plus SM buffer) and negative controls (TSB only) were included. The plate was incubated statically at 37 °C for 24 h to allow biofilm formation. After incubation, the liquid was discarded, and the wells were gently washed three times with PBS and air-dried at room temperature. Each well was stained with 200 µL of 1% crystal violet solution for 15 min. After staining, the crystal violet solution was discarded, and the wells were rinsed and air-dried. The bound crystal violet was dissolved in 200 µL of absolute ethanol, and the absorbance at 600 nm (OD_600_) was measured using a microplate reader.

#### 2.10.3 Clearance of pre-formed biofilms

First, 24 h MRSA biofilms were formed in 96-well plates as described above. After discarding the culture medium, the wells were gently washed three times with PBS to remove planktonic bacteria. For the test groups, 200 µL of phage SPD suspension at different concentrations (10^4^ to 10^8^ PFU/mL) was added, whereas the control groups received an equal volume of SM buffer. The plate was further incubated statically at 37 °C for 18 h. The inhibitory effect of the phage on biofilms was assessed using crystal violet staining, and the clearance rate was calculated accordingly.

### 2.11 Statistical analysis

All experiments were independently performed in triplicate. Data are presented as mean ± standard deviation (SD). Statistical analysis was performed using IBM SPSS Statistics 27 software. Differences between groups were analyzed using one-way analysis of variance (ANOVA), with a P < 0.05 considered statistically significant. Graphs were generated using GraphPad Prism 8 software and the ChiPlot online platform.

## 3 Results

### 3.1 Morphology of bacteriophage SPD

**Figure 1:**
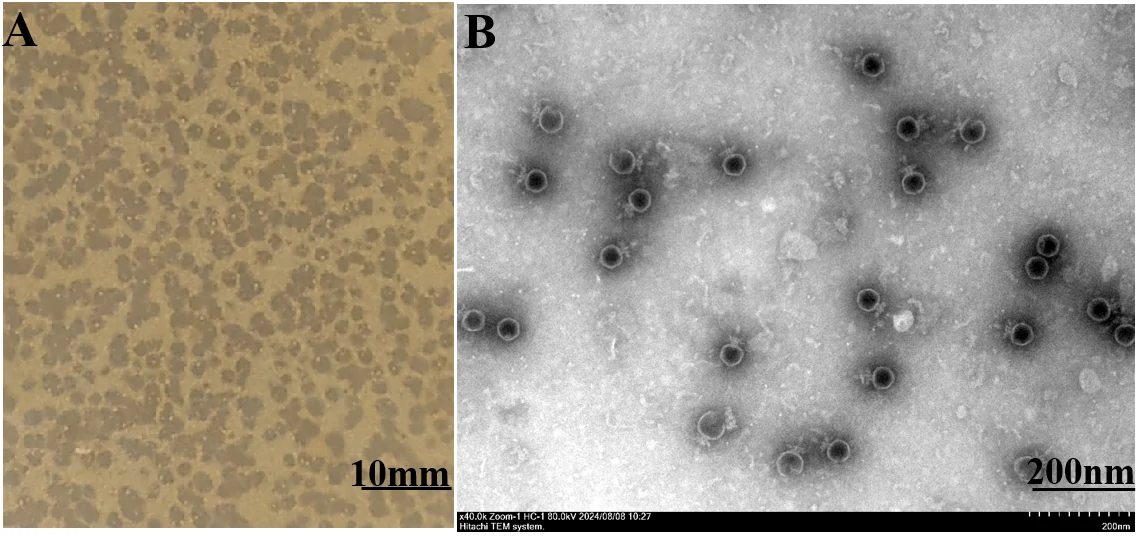
Plaque and morphological characterization of phage SPD. **(A)** Clear plaques (~3 mm in diameter) formed on LB agar plates. **(B)** Transmission electron micrograph of negatively stained phage SPD particles. Scale bar = 200 nm.

Following incubation at 37 °C for 8 h on double-layer agar plates, phage SPD formed clear, transparent plaques with distinct borders, measuring approximately 3 mm in diameter (**Figure 1A**). Transmission electron microscopy analysis revealed that SPD exhibits typical *Podovirus* morphology, characterized by an icosahedral head (~50 nm in diameter) and a short tail phage (~60 nm in length) (**Figure 1B**).

### 3.2 Host range

The host range of phage SPD, determined by the spot assay, is summarized in **Table 1**. Among the 50 tested bacterial strains, SPD exhibited lytic activity against 38 strains, corresponding to an overall lytic rate of 76.0%. Specifically, SPD lysed 29 out of 41 *Staphylococcus aureus* strains (70.7%), encompassing 14 different *spa* types (e.g., t091, t1059, t127, t1670, t2484, t267, t309, t4792, t7229, t2616, t1328, t1736, t189, and t2592). All 7 tested MRSA strains and 2 *Staphylococcus lentus* strains were susceptible to SPD. The susceptible strains were isolated from various animal hosts, including pigeons, sheep, cattle, dogs, and pigs.

### 3.3 Optimal MOI

**Figure 2:**
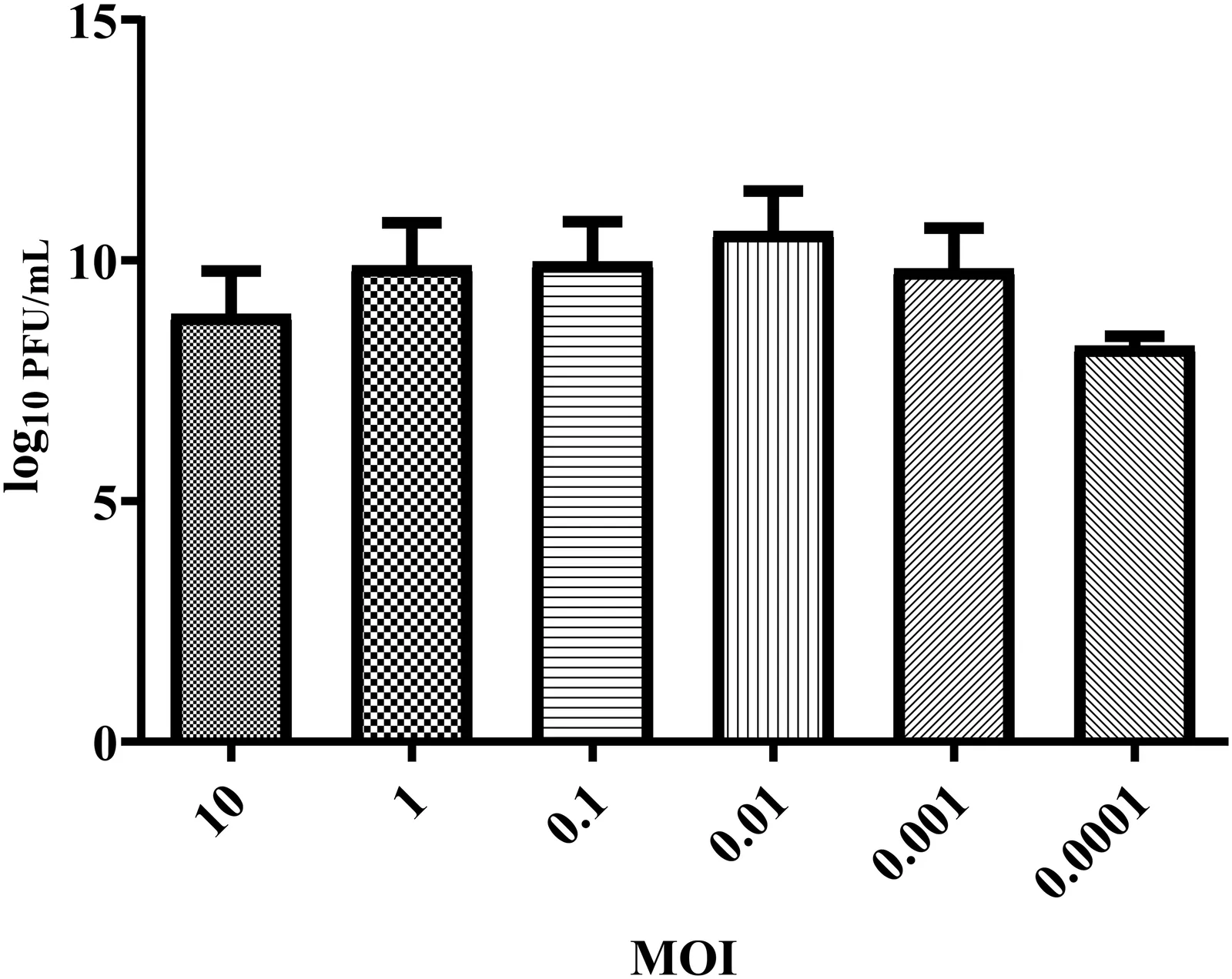
Determination of the optimal MOI for phage SPD. The phage titer was highest at an MOI of 0.01, which was defined as the optimal MOI.

The effect of different MOIs on the propagation efficiency of phage SPD was assessed (**Figure 2**). The phage titer reached a peak of 1.48 × 10¹¹ PFU/mL at an MOI of 0.01. Thus, the optimal MOI for SPD was determined to be 0.01.

### 3.4 Stability of the phage

**Figure 3:**
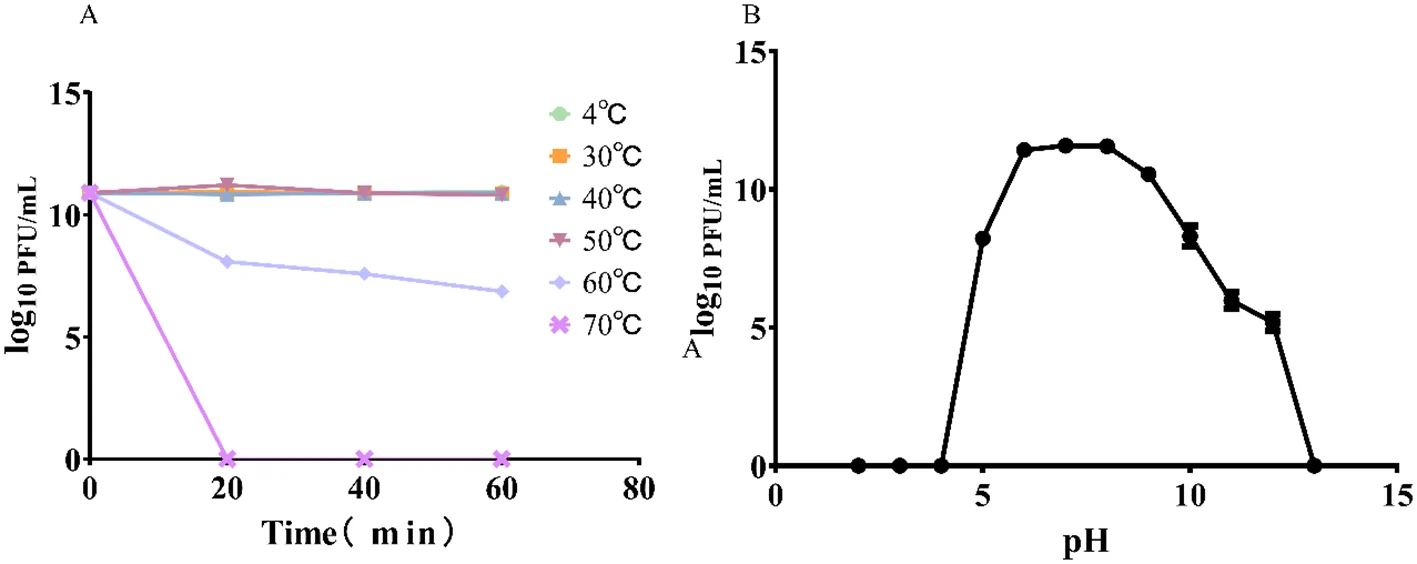
Stability of phage SPD under various conditions. **(A)** Thermal stability. Phage titers remained stable after exposure to temperatures from 4 °C to 50 °C for 1 h. The titer decreased significantly at 60 °C and no viable phage was detected at 70 °C. **(B)** pH stability. The phage titer remained stable in the pH range of 6–9, while complete inactivation occurred at pH < 5 or pH > 12.

As shown in **Figure 3A**, phage SPD maintained stable titers after 60 min of incubation at temperatures ranging from 4 to 50 °C. A significant decrease in titer was observed at 60 °C, although viable phages remained detectable after 60 min of exposure. No viable phage particles were detected following exposure to 70 °C for 20 min. The pH stability assay indicated that phage SPD retained stable activity within a pH range of 6 to 9 (**Figure 3B**). The phage was completely inactivated at pH values below 5 or above 12, indicating a defined pH stability range.

### 3.5 One-step growth curve

**Figure 4:**
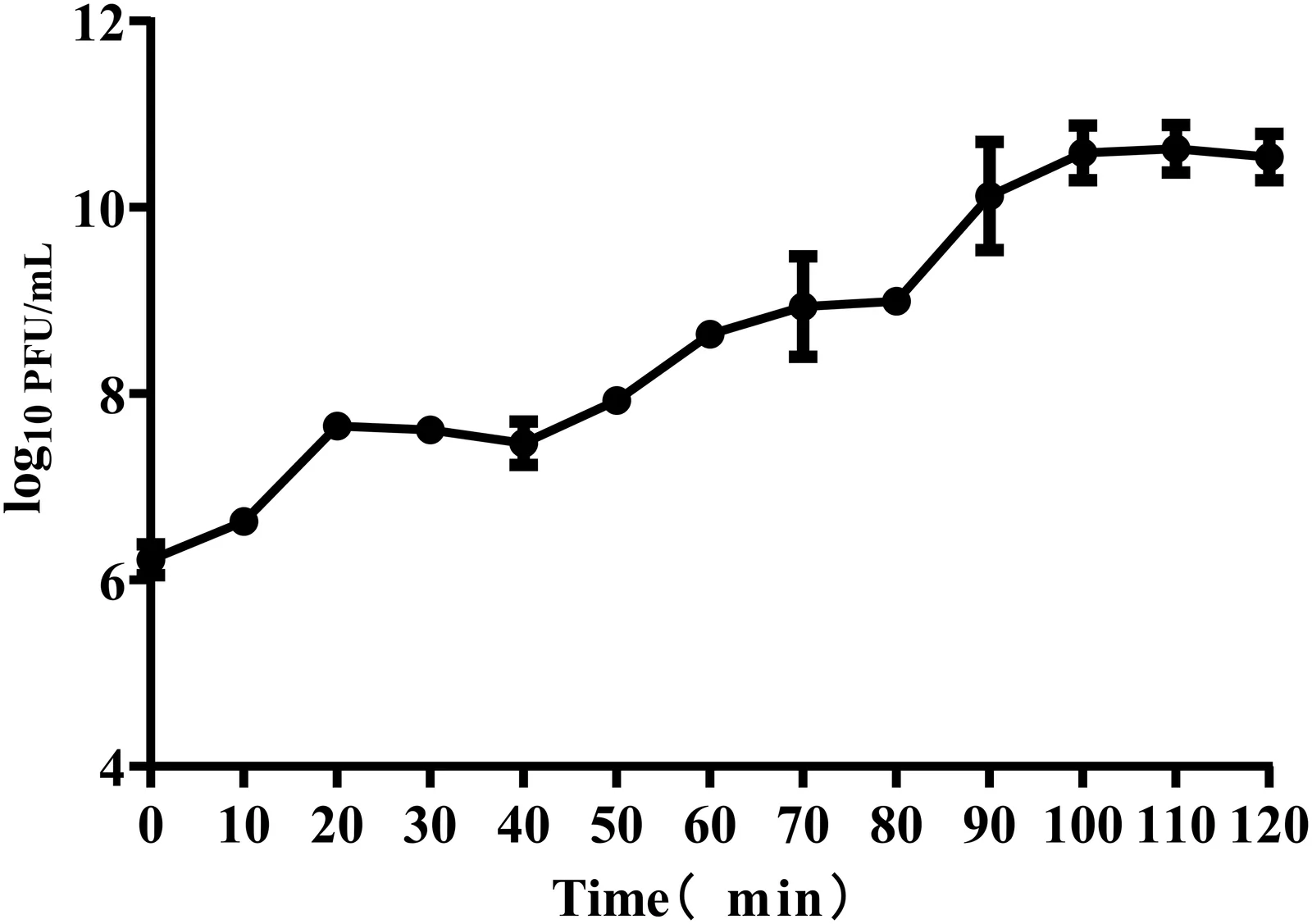
One-step growth curve of phage SPD. The latent period, burst period, and burst size are indicated.

The one-step growth curve revealed the replication kinetics of SPD (**Figure 4**). A latent period of approximately 10 min was observed, followed by a rapid burst phase from 10 to 90 min, resulting in a burst size of 4,470 PFU/cell. The phage titer reached a plateau after 90 min, indicating the completion of the replication cycle.

### 3.6 Genomic features of phage SPD

**Figure 5:**
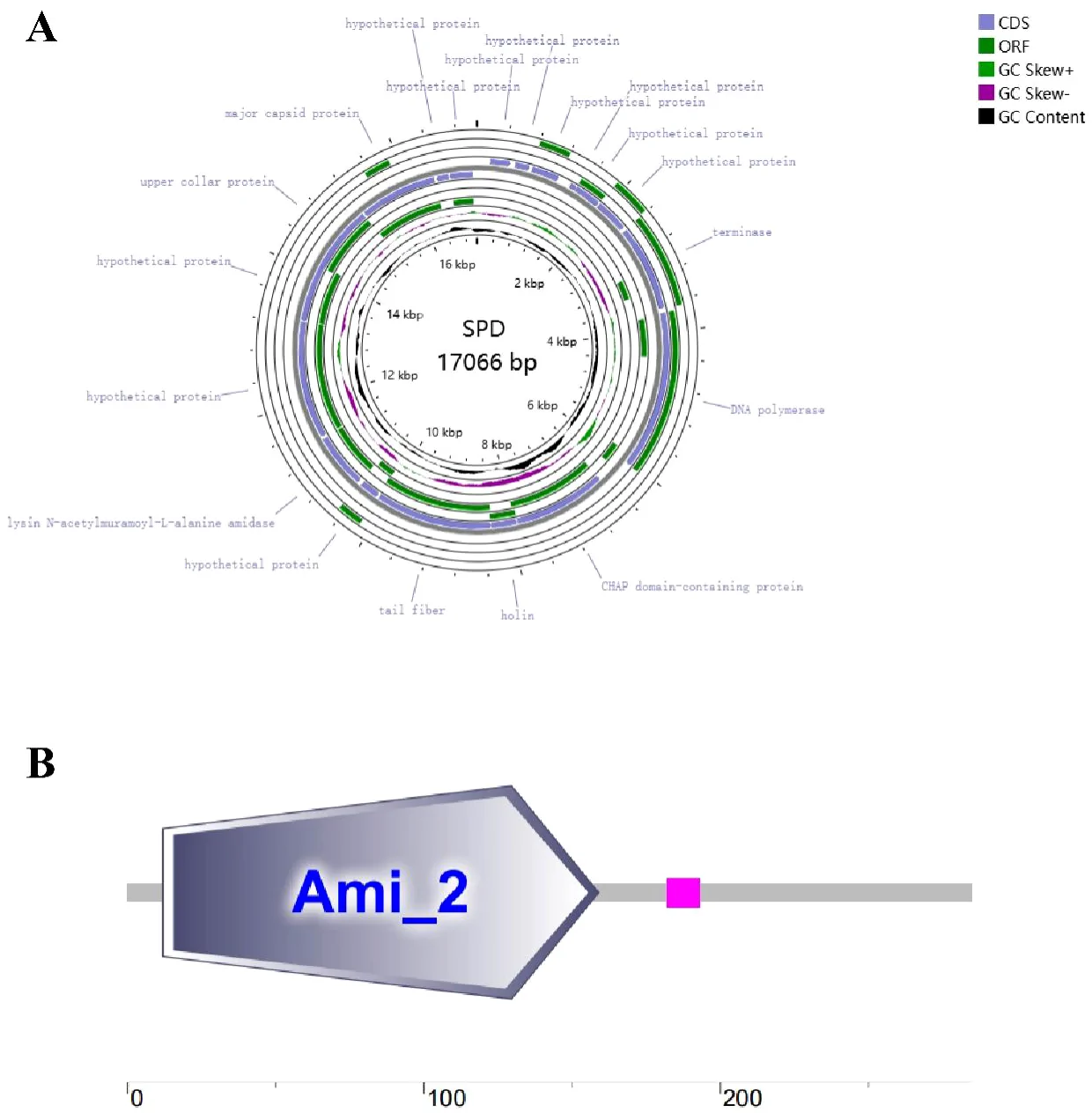
Genomic characterization of phage SPD. **(A)** Circular genome map generated by CGview, illustrating CDS distribution and GC content. **(B)** Domain architecture of the predicted lysin protein analyzed using the SMART tool.

Phage SPD possesses a dsDNA genome of 17,066 bp with a GC content of 35.1% (GenBank accession number: PV358730). The genome structure is shown in **Figure 5A**. Prokka annotation predicted 19 coding sequences (CDSs), with 8 located on the positive strand and 11 on the negative strand. Functional annotation revealed that 7 CDSs encode known functional proteins, including a lysin (N-acetylmuramoyl-L-alanine amidase) and a holin. The remaining 12 CDSs encode hypothetical proteins. No tRNA genes were identified in the genome. Lifestyle prediction using PhageScope classified SPD as a virulent phage, and no anti-CRISPR genes or proteins were detected. Comparative analysis against the VFDB database confirmed the absence of virulence factors and antibiotic resistance genes. Domain analysis of the predicted lysin (CDS13) revealed an Amidase_2 domain (amino acids 12–159), which belongs to the N-acetylmuramoyl-L-alanine amidase family and is involved in hydrolyzing the amide bond of peptidoglycan (**Figure 5B**).

### 3.7 Bioinformatic analysis of the phage genome

#### 3.7.1 Comparative genomic analysis

**Figure 6:**
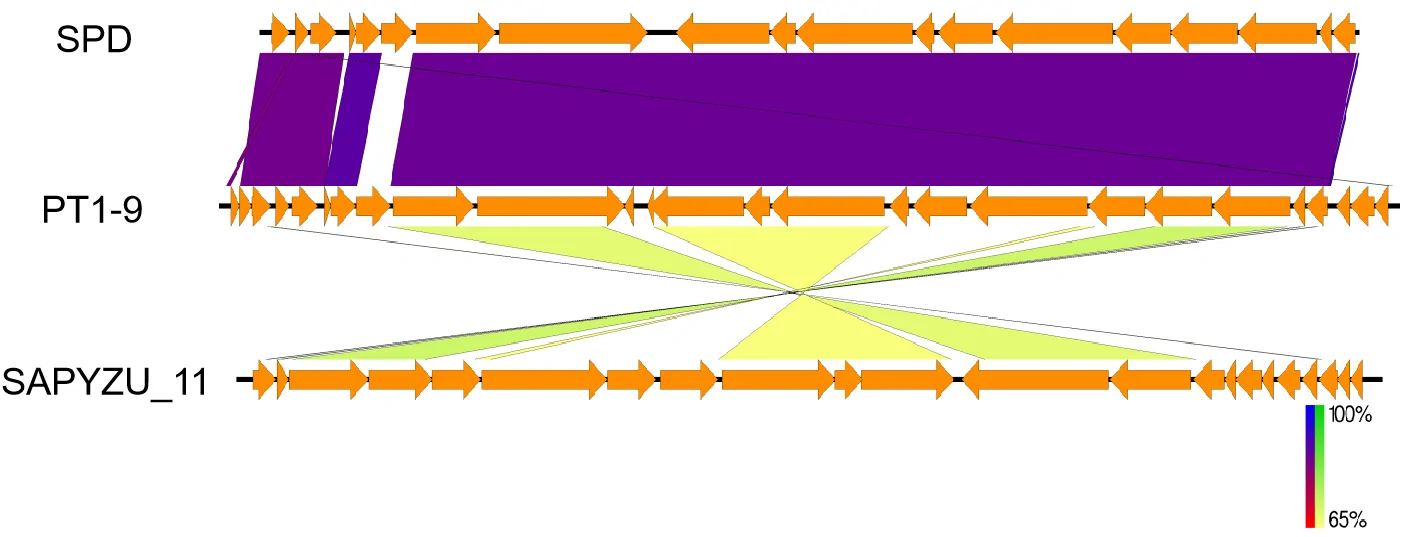
Comparative genomic analysis of phage SPD with related phages PT1–9 and SAPYZU_11. Synteny analysis was performed using EasyFig software (tBLASTx mode).

BLASTn comparison of the SPD genome against the NCBI database revealed high homology with staphylococcal phages belonging to the genus *Andhravirus*. Genomic collinearity analysis was performed with two closely related phages, PT1–9 and SAPYZU_11 (**Figure 6**). The results showed that SPD shares a similar genomic backbone with PT1–9 but exhibits local sequence variations. More pronounced differences were observed in comparison with SAPYZU_11, confirming that SPD represents a novel phage isolate.

#### 3.7.2 Phylogenetic analysis

**Figure 7:**
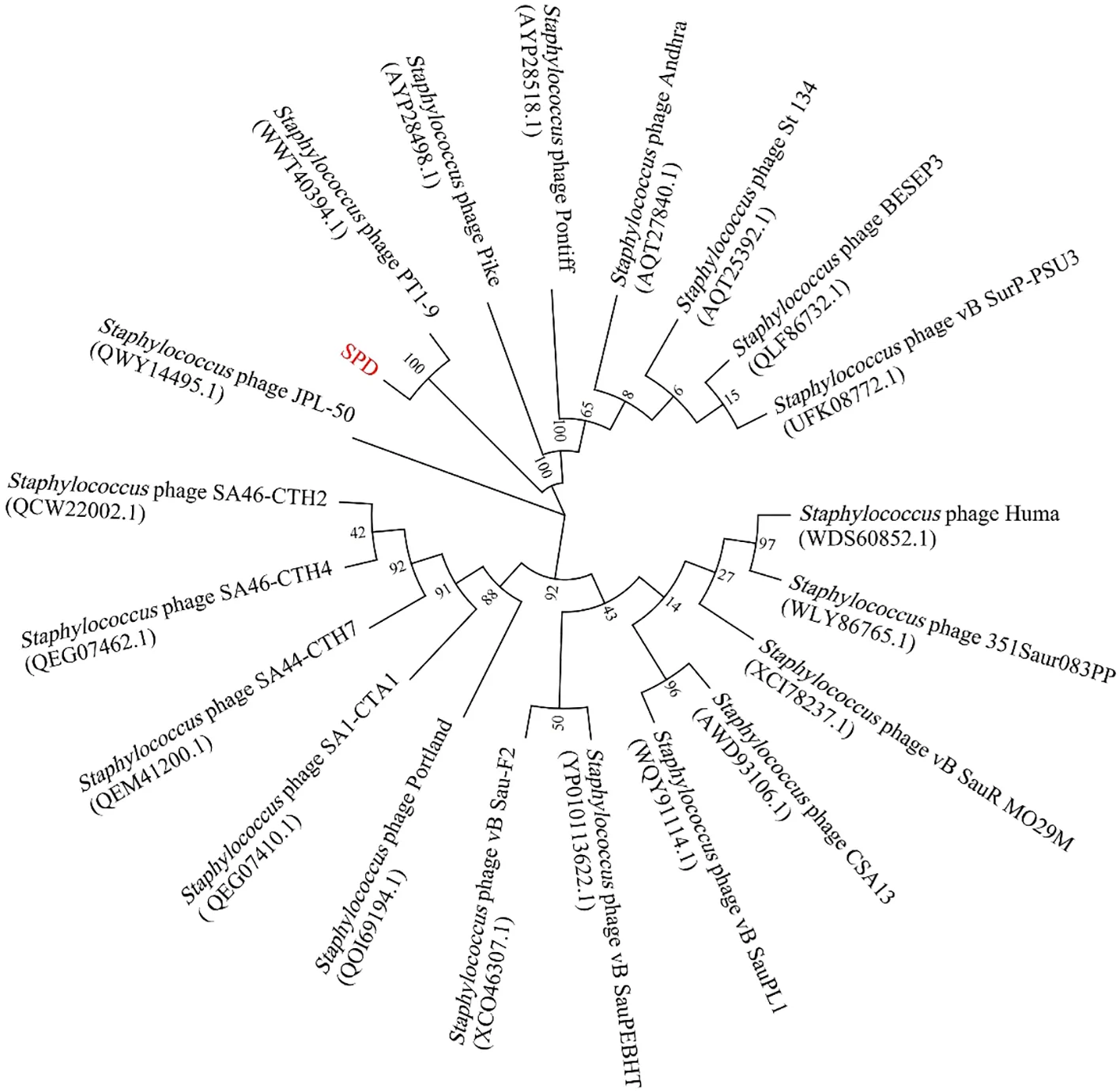
Phylogenetic analysis based on the amino acid sequences of the major capsid protein. The tree was constructed using the neighbor-joining method with 1,000 bootstrap replicates. Phage SPD is highlighted.

A phylogenetic tree was constructed based on the amino acid sequence of the major capsid protein (**Figure 7**). Evolutionary analysis revealed that phage SPD clusters within the same clade as *Andhravirus* phages PT1–9 and Pike, further supporting its classification within the genus *Andhravirus*.

### 3.8 *In vitro* antibacterial efficacy of phage against MRSA

#### 3.8.1 Inhibition of planktonic bacteria at different MOIs

**Figure 8:**
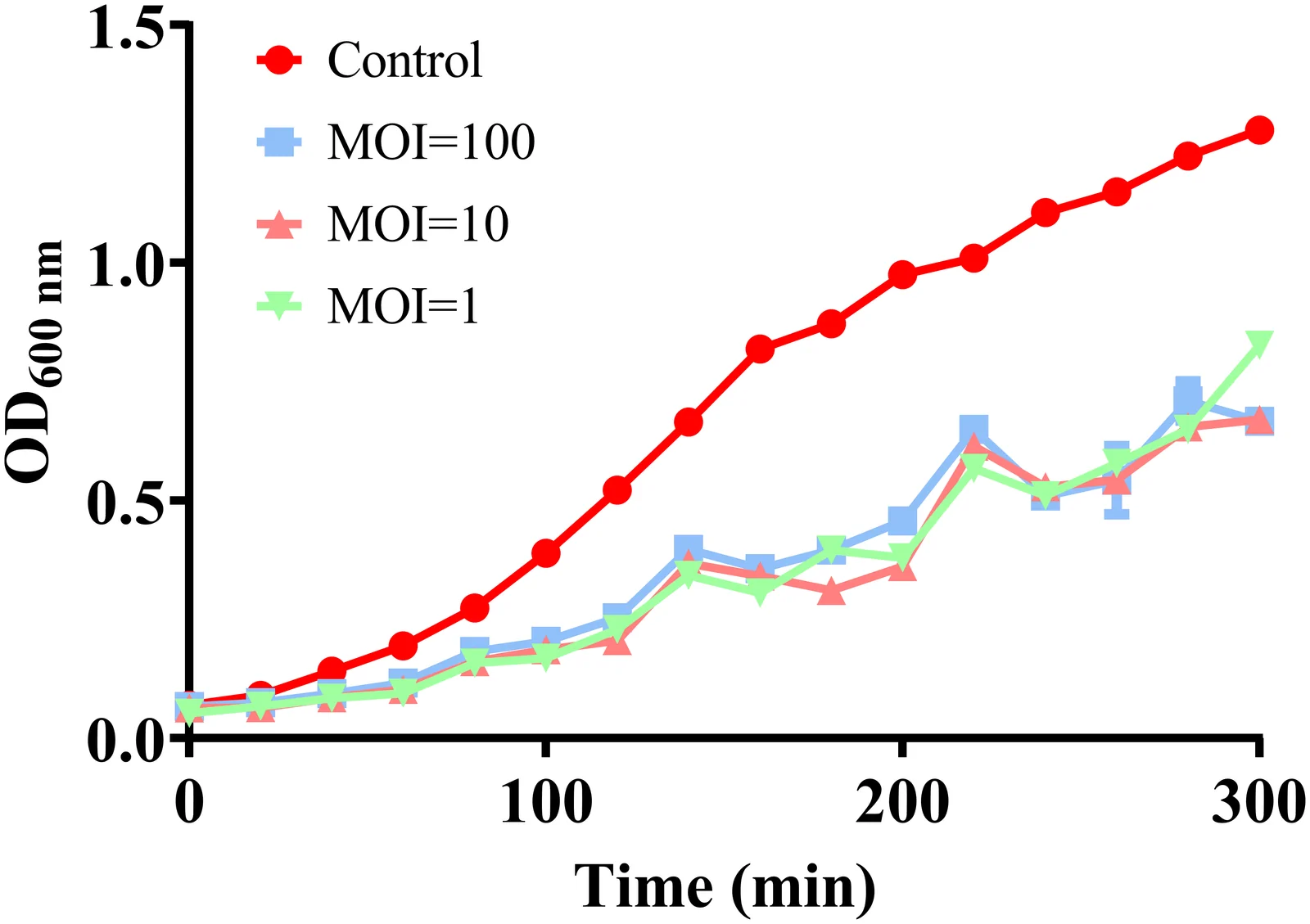
Inhibitory effect of phage SPD at different MOIs on the planktonic growth of MRSA 380-2. Bacterial growth was monitored by measuring OD_600_ nm.

The inhibitory effect of SPD on the planktonic growth of the host strain MRSA 380–2 was assessed by monitoring the OD_600_ nm over time (**Figure 8**). Compared to the control group, SPD at MOIs of 1, 10, and 100 significantly inhibited bacterial growth within 300 min. However, a gradual increase in OD_600_ nm was observed in all treatment groups after 60 min, indicating incomplete bacterial clearance and a decline in inhibitory efficacy over time.

#### 3.8.2 Inhibition of biofilm formation

**Figure 9:**
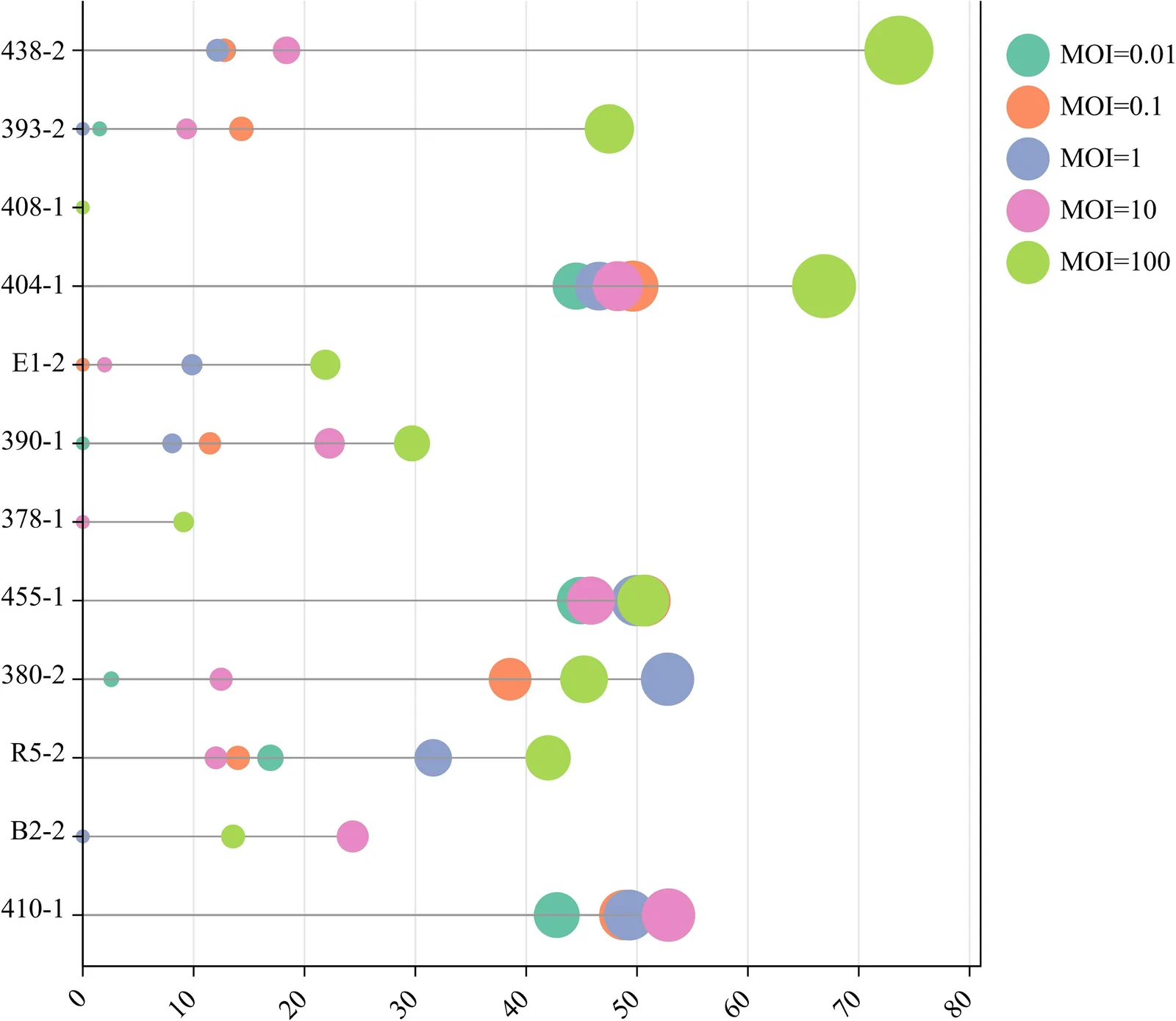
Inhibition rates of biofilm formation by MRSA strains treated with phage SPD at different MOIs.

The inhibitory effect of SPD at different MOIs on biofilm formation by 12 MRSA strains was evaluated (**Figure 9**). SPD inhibited biofilm formation by more than 50% for strains 438-2, 404-1, 455-1, 380-2, and 410-1. For the remaining strains, the inhibition rate was below 30%. The inhibitory effect exhibited concentration-dependency, with higher phage concentrations resulting in greater inhibition.

#### 3.8.3 Eradication of pre-formed mature biofilms

**Figure 10:**
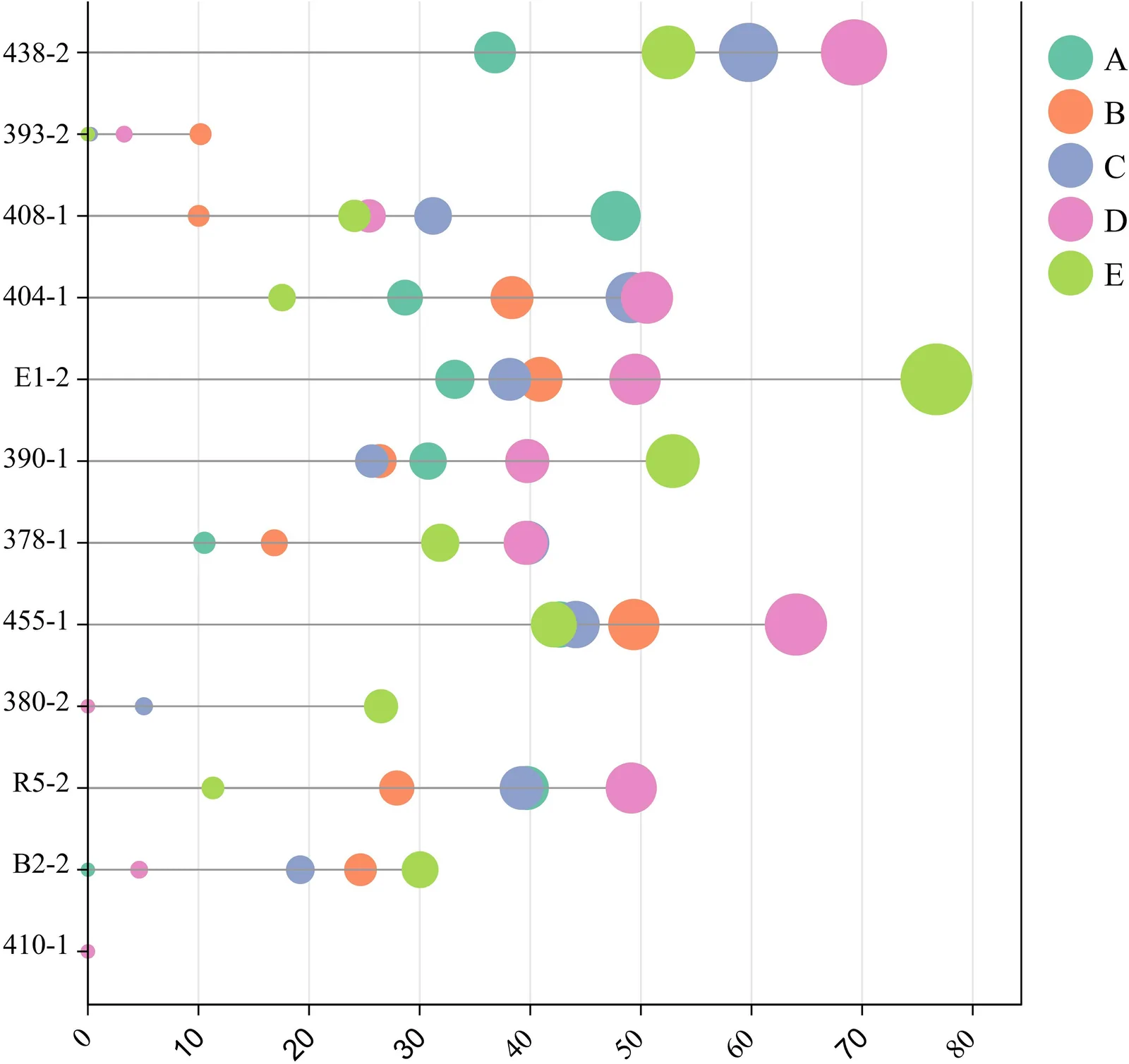
Clearance rates of mature MRSA biofilms treated with phage SPD at different concentrations. Groups A to E represent treatments with phage concentrations of 10^4^, 10^5^, 10^6^, 10^7^, and 10^8^ PFU/mL, respectively.

The ability of SPD at different concentrations to eradicate 24 h mature biofilms was assessed (**Figure 10**). SPD achieved a clearance rate exceeding 60% for strains 438-2, E1-2, and 455-1. For strains 404–1 and 390-1, the clearance rates were 50% and 52%, respectively. For the remaining strains, the clearance rate was below 40%. The eradication efficacy also demonstrated concentration-dependency, with higher phage concentrations correlating with stronger clearance activity.

## 4 Discussion

In this study, a novel *Staphylococcus* phage, designated SPD, was successfully isolated and characterized from wastewater collected at a pigeon farm in Kashgar, Xinjiang. Transmission electron microscopy revealed that SPD exhibits typical *Podovirus* morphology, which is consistent with common morphological types of staphylococcal phages (Cho et al., 2025). One-step growth curve analysis indicated that SPD exhibits a latent period of approximately 10 min and a high burst size of 4,470 PFU/cell, demonstrating efficient replicative capacity-a key indicator for assessing therapeutic potential (Wen et al., 2023; Zhou et al., 2023). Its optimal MOI was 0.01, suggesting that efficient lysis can be achieved at a low initial phage concentration. This finding provides valuable insight for optimizing phage preparation production and clinical application strategies.

SPD displayed a relatively broad host range, lysing 76.0% of the tested strains, with notable activity against various animal-derived *S*. aureus isolates spanning 14 *spa* types and all targeted MRSA strains (7/7). This indicates the potential of SPD to target prevalent staphylococcal strains within a specific ecological niche, such as the poultry farming environment. However, limitations in its lytic spectrum were also observed, including the inability to lyse certain common healthcare-associated MRSA lineages. This underscores the necessity of developing phage cocktail therapies to overcome the host range limitations of a single phage (Pelyuntha et al., 2025).

Stability is a crucial factor for the storage and application of potential therapeutic agents. SPD remained stable within a temperature range of 4-50 °C and a pH range of 6-9, indicating good tolerance under routine physiological and storage conditions (Mohammadian et al., 2022; Fernandez et al., 2025). Nevertheless, its inactivation under extreme temperatures and pH conditions highlights the need for stringent control of environmental parameters during production and application.

Whole-genome analysis is central to evaluating the safety of phages. The SPD genome is a double-stranded DNA molecule 17,066 bp in length with a GC content of 35.1%. Its size and composition are similar to those of known phages within the genus *Andhravirus (*Chavignon et al., 2022; Kwon et al., 2022; Golosova et al., 2024). Bioinformatic analysis detected no known virulence factors, antibiotic resistance genes, or anti-CRISPR proteins in its genome, preliminarily eliminating the risk of horizontal gene transfer of harmful traits (Guo et al., 2024; Raveendran et al., 2026). Functional annotation identified key components for lytic activity, namely an N-acetylmuramoyl-L-alanine amidase (lysin) and a holin. The lysin, in particular, represents a promising candidate for future development as a standalone anti-biofilm agent (Asadi et al., 2023; Vazquez et al., 2025; Wen et al., 2025; Gao et al., 2026).

Biofilm formation by MRSA is a key factor leading to chronic infections and treatment failure (Totten and Patel, 2022; Dakheel et al., 2025). *In vitro* assays in this study confirmed that phage SPD not only inhibited the growth of planktonic MRSA but also effectively prevented biofilm formation (inhibition >50% for some strains) and eradicated pre-formed mature biofilms (clearance >60% for some strains) in a concentration-dependent manner. These findings align with the reported mechanisms of many phages, which can directly degrade the extracellular matrix via depolymerases or lysins and persistently infect bacteria within the biofilm structure (Kim et al., 2025; Li et al., 2025).

However, we observed that the inhibitory effect of SPD on planktonic bacteria diminished over time, and its efficacy in eradicating biofilms varied among different strains. This variability may be attributed to heterogeneous phage penetration due to the physical barrier of the biofilm matrix, bacterial heterogeneity (e.g., the emergence of persister cells), or the development of phage tolerance (Bleick et al., 2025; Krusche et al., 2025). These observations suggest that future applications of SPD for treating biofilm-associated infections may require combination strategies with other agents, such as low-dose antibiotics, enzymes, or physical methods like electroporation, to enhance penetration and synergistic effects (Parmar et al., 2025).

Phylogenetic analysis based on the major capsid protein sequence, along with whole-genome collinearity comparison with *Andhravirus* phages PT1–9 and SAPYZU_11, definitively classified SPD within the genus *Andhravirus*. While SPD shares a similar genomic backbone with PT1-9, local sequence variations indicate they likely share a common ancestor but have diverged during evolution, possibly adapting to distinct host microenvironments (Li et al., 2024). These findings contribute new material to the study of evolutionary relationships and functional genomics among staphylococcal phages.

## 5 Conclusion

In this study, we successfully isolated a novel virulent *Staphylococcus* phage, designated SPD, from wastewater collected at a pigeon farm in Kashgar, Xinjiang. Morphological characterization confirmed SPD as a *Podovirus* with a broad host range (lytic rate of 76.0%), a short latent period (~10 min), a high burst size (~4,470 PFU/cell), and favorable thermal and pH stability. Genomic analysis revealed that SPD is a dsDNA virus belonging to the genus *Andhravirus*, lacking virulence factors, antibiotic resistance genes, and anti-CRISPR genes. *In vitro* experiments demonstrated that SPD effectively inhibits planktonic MRSA growth and exhibits concentration-dependent inhibition of biofilm formation as well as eradication of mature biofilms. Collectively, this study provides a safe and effective phage candidate and presents experimental evidence supporting the development of phage-based therapy against animal-derived MRSA and its biofilm-associated infections, particularly in the Xinjiang region.

## Data Availability

The datasets presented in this study can be found in online repositories. The names of the repository/repositories and accession number(s) can be found in the article/supplementary material.

## References

[B1] Asadi M. Taheri-Anganeh M. Ranjbar M. Khatami S. H. Maleksabet A. Mostafavi-Pour Z. . (2023). LYZ2-SH3b as a novel and efficient enzybiotic against methicillin-resistant Staphylococcus aureus. BMC Microbiol. 23, 257. doi: 10.1186/s12866-023-03002-9 37704938 PMC10500863

[B2] Bleick C. R. Van Helden S. R. Berti A. D. Richa R. Lehman S. M. Lehman S. M. . (2025). Optimizing phage-antibiotic combinations: impact of administration order against daptomycin non-susceptible (DNS) MRSA clinical isolates. Antimicrob. Agents Chemother. 69, e0069925. doi: 10.1128/aac.00699-25 41251374 PMC12691696

[B3] Ceballos-Zuniga F. Galvez-Larrosa L. Munoz I. G. Infantes L. Fernandez-Carrillo J. Perez-Dorado I. (2025). Dissecting the molecular basis underlying mycobacterial cell-wall hydrolysis by the catalytic domains of D29LysA and DS6ALysA phage endolysins. Int. J. Biol. Macromol. 334, 148896. doi: 10.1016/j.ijbiomac.2025.148896 41207583

[B4] Chavignon M. Kolenda C. Medina M. Bonhomme M. Blazere L. Legendre T. . (2022). Bacteriophage-based decontamination to control environmental colonization by Staphylococcus capitis in neonatal intensive care units: An *in vitro* proof-of-concept. Front. Cell. Infect. Microbiol. 12, 1060825. doi: 10.3389/fcimb.2022.1060825 36467721 PMC9710537

[B5] Cho J. H. Lee G. M. Ko S. Kim Y. Kim D. (2025). Characterization and therapeutic potential of newly isolated bacteriophages against Staphylococcus species in bovine mastitis. J. Virol. 99, e0190124. doi: 10.1128/jvi.01901-24 39950776 PMC11915829

[B6] Dakheel K. H. Rahim R. A. Al-Obaidi J. R. Razali N. Neela V. K. Hun T. G. . (2025). Proteomic analysis reveals phage-driven metabolic shifts and biofilm disruption in methicillin-resistant Staphylococcus aureus (MRSA). World J. Microbiol. Biotechnol. 41, 230. doi: 10.1007/s11274-025-04397-5 40560268 PMC12198068

[B7] Doud M. B. Robertson J. M. Strathdee S. A. (2025). Optimizing phage therapy with artificial intelligence: a perspective. Front. Cell. Infect. Microbiol. 15, 1611857. doi: 10.3389/fcimb.2025.1611857 40496019 PMC12149144

[B8] Duarte A. C. Fernandez L. Rodriguez A. Garcia P. (2025). A new bacteriophage infecting Staphylococcus epidermidis with potential for removing biofilms by combination with chimeric lysin CHAPSH3b and vancomycin. mSphere 10, e0101424. doi: 10.1128/msphere.01014-24 39982075 PMC11934314

[B9] Fernandez L. Duarte A. C. Jurado A. Bueres L. Rodriguez A. Garcia P. (2025). Multipronged impact of environmental temperature on Staphylococcus aureus infection by phage Kayvirus rodi: Implications for biofilm control. Biofilm 9, 100248. doi: 10.1016/j.bioflm.2024.100248 39845530 PMC11751508

[B10] Gao M. Yu X. Yang C. Mi Z. Bai C. Liu C. . (2026). Characteristics and antibacterial activity of Staphylococcus aureus phage-derived endolysin LysP4. Probiotics Antimicrob. Proteins 18, 681–693. doi: 10.1007/s12602-025-10543-0 40299201

[B11] Garcia-de-Albeniz N. Muller D. W. Mucklich F. Ginebra M. P. Jimenez-Pique E. Mas-Moruno C. (2026). Multiscale hierarchical surface structuring of zirconia using femtosecond laser and chemical etching: Implications for cell response and antibacterial performance. Mater. Today Bio 36, 102648. doi: 10.1016/j.mtbio.2025.102648 41497901 PMC12767873

[B12] Golosova N. N. Matveev A. L. Tikunova N. V. Khlusevich Y. A. Kozlova Y. N. Morozova V. V. . (2024). Bacteriophage vB_SepP_134 and endolysin LysSte_134_1 as potential Staphylococcus-biofilm-removing biological agents. Viruses 16, 385. doi: 10.3390/v16030385 38543751 PMC10975630

[B13] Guo M. Zhang Y. Wu L. Xiong Y. Xia L. Cheng Y. . (2024). Development and mouse model evaluation of a new phage cocktail intended as an alternative to antibiotics for treatment of Staphylococcus aureus-induced bovine mastitis. J. Dairy Sci. 107, 5974–5987. doi: 10.3168/jds.2024-24540 38522833

[B14] Kim J. Liao X. Zhang S. Ding T. Ahn J. (2025). Application of phage-derived enzymes for enhancing food safety. Food Res. Int. 209, 116318. doi: 10.1016/j.foodres.2025.116318 40253159

[B15] Krusche J. Beck C. Lehmann E. Gerlach D. Daiber E. Mayer C. . (2025). Characterization and host range prediction of Staphylococcus aureus phages through receptor-binding protein analysis. Cell Rep. 44, 115369. doi: 10.1016/j.celrep.2025.115369 40022731

[B16] Kwon H. Park S. Y. Kim M. S. Kim S. G. Park S. C. Kim J. H. (2022). Characterization of a lytic bacteriophage vB_SurP-PSU3 infecting Staphylococcus ureilyticus and its efficacy against biofilm. Front. Microbiol. 13, 925866. doi: 10.3389/fmicb.2022.925866 35923398 PMC9340203

[B17] Li X. Zhang B. Tong X. Zhou T. Li M. Barkema H. W. . (2024). Biological and genomic characterization of 4 novel bacteriophages isolated from sewage or the environment using non-aureus Staphylococci strains. Vet. Microbiol. 294, 110133. doi: 10.1016/j.vetmic.2024.110133 38820726

[B18] Li Y. Dai J. Wu S. Rong D. Huang J. Zhao M. . (2025). The food application of a novel Staphylococcus aureus bacteriophage vB_SA_STAP152 and its endolysin LysP152 with high enzymatic activity under cold temperature. Food Microbiol. 128, 104710. doi: 10.1016/j.fm.2024.104710 39952757

[B19] Liu X. Da X. Wu Y. Xu Y. Shi Y. Wu A. . (2026). Eradicating extra- and intra-cellular Staphylococcus aureus and MRSA with little accumulation of resistance by a cationic BODIPY derivative. Eur. J. Med. Chem. 303, 118481. doi: 10.1016/j.ejmech.2025.118481 41397327

[B20] Liu Y. Zheng X. Xu L. Tong P. Zhu M. Peng B. . (2021). Prevalence, antimicrobial resistance, and molecular characterization of Staphylococcus aureus isolated from animals, meats, and market environments in Xinjiang, China. Foodborne Pathog. Dis. 18, 718–726. doi: 10.1089/fpd.2020.2863 33534639

[B21] Ma L. Liu Y. Zheng X. Zheng B. Cheng Y. Cai Y. . (2025). Isolation and characterization of methicillin-resistant Staphylococcus aureus phage SPB against MRSA planktonic cells and biofilm. Front. Microbiol. 16, 1554182. doi: 10.3389/fmicb.2025.1554182 40469720 PMC12134071

[B22] Mohammadian F. Rahmani H. K. Bidarian B. Khoramian B. (2022). Isolation and evaluation of the efficacy of bacteriophages against multidrug-resistant (MDR), methicillin-resistant (MRSA) and biofilm-producing strains of Staphylococcus aureus recovered from bovine mastitis. BMC Vet. Res. 18, 406. doi: 10.1186/s12917-022-03501-3 36384653 PMC9670557

[B23] Mulani M. S. Kumkar S. N. Pardesi K. R. (2022). Characterization of novel Klebsiella phage PG14 and its antibiofilm efficacy. Microbiol. Spectr. 10, e0199422. doi: 10.1128/spectrum.01994-22 36374021 PMC9769620

[B24] Parmar K. Chaudhry W. Fackler J. R. Sowers J. M. Vadlamudi A. Greenwood-Quaintance K. . (2025). *In vitro* activity of phages against periprosthetic joint infection-associated staphylococcal biofilms. Sci. Rep. 15, 22199. doi: 10.1038/s41598-025-08759-9 40596635 PMC12214714

[B25] Pelyuntha W. Wannasrichan W. Yamik D. Y. Khongkhai H. Yingkajorn M. Vongkamjan K. (2025). Exploring two polyvalent lytic Staphylococcus phages and their application in controlling Staphylococcus spp. in plant-based meat. Food Res. Int. 221, 117351. doi: 10.1016/j.foodres.2025.117351 41174427

[B26] Pye H. V. Krishnamurthi R. Cook R. Adriaenssens E. M. (2024). Phage diversity in one health. Essays Biochem. 68, 607–619. doi: 10.1042/ebc20240012 39475220 PMC12055037

[B27] Raveendran K. Sharaf S. D A. L. K R. Joseph T. C. Thangaraj R. S. . (2026). Novel Silviavirus phages with broad-host-range activity against methicillin resistant Staphylococcus aureus from seafood: comprehending the phenotypic and genotypic variability. Folia Microbiol. (Praha) 71, 701–718. doi: 10.1007/s12223-025-01290-4 40629134 PMC13346280

[B28] Santonocito R. Avnet S. Tuccitto N. Musso N. Cavallaro A. Spinello M. . (2026). Rapid detection of Staphylococcus aureus using a fluorescent PoC array for perioperative guidance for infection. Biosens. Bioelectron. 294, 118199. doi: 10.1016/j.bios.2025.118199 41206999

[B29] Totten K. M. C. Patel R. (2022). Phage activity against planktonic and biofilm Staphylococcus aureus periprosthetic joint infection isolates. Antimicrob. Agents Chemother. 66, e0187921. doi: 10.1128/aac.01879-21 34662191 PMC8765226

[B30] Vazquez R. Gutierrez D. Grimon D. Fernandez L. Garcia P. Rodriguez A. . (2025). The new SH3b_T domain increases the structural and functional variability among SH3b-like CBDs from staphylococcal phage endolysins. Probiotics Antimicrob. Proteins 17, 3930–3943. doi: 10.1007/s12602-024-10309-0 39080103 PMC12634807

[B31] Wang D. Wang Z. Yan Z. Wu J. Ali T. Li J. . (2015). Bovine mastitis Staphylococcus aureus: antibiotic susceptibility profile, resistance genes and molecular typing of methicillin-resistant and methicillin-sensitive strains in China. Infect. Genet. Evol. 31, 9–16. doi: 10.1016/j.meegid.2014.12.039 25582604

[B32] Wang H. Zou B. Liu Y. Sun Y. Zhao Y. Liu Y. . (2026). Probing the repair mechanism of ohmic heating-induced sublethal injury in Staphylococcus aureus: Multi-omics analysis uncovers the remodeling patterns of proteins and lipids and applications in simulated food systems. Food Microbiol. 134, 104944. doi: 10.1016/j.fm.2025.104944 41136161

[B33] Wang L. Tkhilaishvili T. Jiang Z. Pirlar R. F. Ning Y. Millan Laleona A. . (2024). Phage-liposome nanoconjugates for orthopedic biofilm eradication. J. Control Release 376, 949–960. doi: 10.1016/j.jconrel.2024.09.049 39384150

[B34] Wen Q. Huang X. Ma W. Chen Y. Wang L. Ma Y. . (2025). Characterization of a phage endolysin LysLFP01 and its antibacterial activity. Int. J. Food Microbiol. 432, 111110. doi: 10.1016/j.ijfoodmicro.2025.111110 39951925

[B35] Wen H. Zhou W. Wu Y. Li Y. Zhu G. Zhang Z. . (2023). Effective treatment of a broad-host-range lytic phage SapYZU15 in eliminating Staphylococcus aureus from subcutaneous infection. Microbiol. Res. 276, 127484. doi: 10.1016/j.micres.2023.127484 37659336

[B36] Zhang R. Stehle Y. Chen L. Zeng K. Lin M. Wang C. . (2026). Multifunctional, enzyme/pH-responsive gelatin microspheres with aptamer-targeted antibacterial and ionic-mediated dual therapy for infected bone defects. Biomaterials 326, 123642. doi: 10.1016/j.biomaterials.2025.123642 40854266

[B37] Zhang F. Wu S. Gao J. Ma S. Pei X. Yang X. . (2026). Application of a novel phage vB_Sa_2868B2 and its endolysin LytN with broad antibacterial spectrum against multidrug-resistant Staphylococcus aureus on food matrices, and metal surfaces. Int. J. Food Microbiol. 448, 111575. doi: 10.1016/j.ijfoodmicro.2025.111575 41406564

[B38] Zhang Q. Xing S. Sun Q. Pei G. Cheng S. Liu Y. . (2017). Characterization and complete genome sequence analysis of a novel virulent Siphoviridae phage against Staphylococcus aureus isolated from bovine mastitis in Xinjiang, China. Virus Genes 53, 464–476. doi: 10.1007/s11262-017-1445-z 28299517

[B39] Zheng X. Liu M. Li P. Xu S. Chen L. Xu G. . (2024). Antibacterial activity evaluation of a novel K3-specific phage against Acinetobacter baumannii and evidence for receptor-binding domain transfer across morphologies. Virol. Sin. 39, 767–781. doi: 10.1016/j.virs.2024.08.002 39098716 PMC11738781

[B40] Zheng X. Wang X. Li P. Zhou Y. Zhu X. Hu Z. . (2025). The change of long tail fibers expanded the host range of a T5-like Salmonella phage and its application in milk. BMC Microbiol. 25, 169. doi: 10.1186/s12866-025-03895-8 40133802 PMC11938639

[B41] Zhou W. Y. Wen H. Li Y. J. Gao Y. J. Zheng X. F. Li H. X. . (2023). WGS analysis of two Staphylococcus aureus bacteriophages from sewage in China provides insights into the genetic feature of highly efficient lytic phages. Microbiol. Res. 271, 127369. doi: 10.1016/j.micres.2023.127369 36996644

[B42] Zhu G. S. Yang X. X. Zhang Y. Zhang X. Y. Lin L. B. Wang F. (2026). Bacteriophage lysin P26ly combats multidrug-resistant Escherichia coli O157 and methicillin-resistant Staphylococcus aureus via cell wall degradation and membrane disruption. Int. J. Biol. Macromol. 336, 149288. doi: 10.1016/j.ijbiomac.2025.149288 41317755

